# Aspirin in patients with glucose-6-phosphate dehydrogenase deficiency: a true clinical issue?

**DOI:** 10.1093/ehjcvp/pvae101

**Published:** 2024-12-31

**Authors:** Gianmarco Sarto, Emanuele Soraci, Sebastiano Sciarretta, Mattia Galli

**Affiliations:** Department of Medical Surgical Sciences and Biotechnologies, Sapienza University of Rome, Corso della Repubblica 79, 04100 Latina, Italy; ASP 5—Ospedale di Patti, Messina, Italy; Department of Medical Surgical Sciences and Biotechnologies, Sapienza University of Rome, Corso della Repubblica 79, 04100 Latina, Italy; IRCCS Neuromed, Pozzilli, IS, Italy; Department of Medical Surgical Sciences and Biotechnologies, Sapienza University of Rome, Corso della Repubblica 79, 04100 Latina, Italy; Maria Cecilia Hospital, GVM Care & Research, Via Corriera 1, 48033, Cotignola, Italy

Glucose-6-phosphate dehydrogenase (G6PD) deficiency is the most common enzyme defect, affecting over 500 million people worldwide.^1^ G6PD is an X-linked hereditary condition caused by mutations in the G6PD gene, resulting in a wide range of enzyme activity levels and a spectrum of clinical phenotypes.^1^ G6PD is a key enzyme in the pentose phosphate pathway, responsible for producing nicotinamide adenine dinucleotide phosphate (NADPH), which protects cells against oxidative stress.^1^ In red blood cells, which lack mitochondria, NADPH acts as the primary defence mechanism against oxidative damage.^1^ Consequently, G6PD deficiency makes red blood cells highly susceptible to oxidative stress, manifesting as neonatal jaundice or acute haemolytic anaemia, often triggered by infections, drugs, or certain foods like fava beans. Preventing oxidative stress remains the cornerstone of management.

Several drugs are known to cause dangerous to severe haemolysis in individuals with G6PD deficiency. Others, such as aspirin, are classified as potential oxidizing agents.^1^

In patients with cardiovascular diseases (CVD), low-dose of aspirin represents a cornerstone of treatment due to its antiplatelet effects.^2^ The administration of low-dose aspirin in individuals with CVD and concomitant G6PD deficiency poses a significant clinical challenge. Current guidelines offer no specific recommendations, and the existing evidence in the literature remains limited.^3^

The first study investigating the use of aspirin in patients with CVD and G6PD deficiency was published over 30 years ago.^4^ In this study, among 44 patients with ischaemic cardiomyopathy treated with aspirin at a dose of 250 mg/day, none exhibited signs of haemolysis, with haemoglobin levels, reticulocyte counts, and bilirubin remaining within normal ranges after 3 months of treatment.^4^ Subsequent research was largely limited to case reports and case series until 2020, when a landmark study in Northern Sardinia—an area with the highest prevalence of G6PD deficiency in the Mediterranean—prospectively enrolled 625 consecutive acute coronary syndrome (ACS) patients undergoing percutaneous coronary intervention (PCI). All the patients were treated with low-dose aspirin (100 mg/day) in accordance with guideline recommendations and had G6PD activity measured.^5^ The prevalence of G6PD deficiency was 9% (56 patients), with severe G6PD deficiency being detected in 7% (*n* = 32) of males and in 0.6% (*n* = 1) of females (*P* < 0.001).^5^ Over a follow-up of 18 ± 9 months, adherence to aspirin was 100% and was not associated with aspirin-induced clinically relevant haemolysis.^5^ One patient died from sepsis and multiorgan failure with possible drug-related haemolysis not related to aspirin.^5^

Although the available evidence remains limited, studies in patients with ischaemic cardiomyopathy collectively indicate that low-dose aspirin is generally well-tolerated and safe.

However, recent concerns about the use of low-dose aspirin in G6PD-deficient patients with CVD have been raised by a group of researchers from China, particularly in the context of patients with a history of ischaemic stroke.^6–8^ In particular, a case of acute haemolysis was reported in one of the 81 G6PD-deficient patients (1.2%) with recent ischaemic stroke treated with low-dose aspirin (100 mg/day) for 3 months.^6^ Similarly, the authors reported a case of late-onset haemolysis and subsequent fatal subdural haemorrhage in a G6PD-deficient individual receiving low-dose aspirin.^8^ The same group of investigators reported that among 279 patients with acute ischaemic stroke (40 G6PD-deficient and 239 G6PD-normal) treated with aspirin at 100 mg/day, the G6PD-deficient group exhibited significantly higher rates of haemoglobin decline ≥25 g/L or 25% from baseline (15.0% vs. 3.3%; *P* = 0.006) and anaemia (30.0% vs. 14.6%; *P* = 0.016), along with a more pronounced increase in bilirubin levels, indicative of haemolysis.^7^

Although these studies originate from a single research group and focus exclusively on Chinese patients, they underscore the importance of exercising caution when prescribing low-dose aspirin to patients with a recent stroke. The limited and non-univocal evidence on the safety and efficacy of low-dose aspirin in G6PD-deficient individuals with CVD underscores the need for dedicated randomized controlled trials. To this extent, the ongoing *Safety and Efficacy of Aspirin in Stroke Patients With Glucose-6-phosphate Dehydrogenase Deficiency* (SAST, NCT04088513) trial will provide important insights.

In the meanwhile, strategies to avoid the use of aspirin in patients with CVD include the use of alternative antiplatelet agents, such as clopidogrel, particularly for patients requiring single antiplatelet therapy. For patients needing dual antiplatelet therapy, such as those with ACS or undergoing PCI, ‘aspirin free’ antiplatelet strategies including the use of P2Y_12_ inhibitor monotherapy after a short (i.e. 1–3 months) course of DAPT is increasingly supported by evidence and may be particularly advantageous in this setting, limiting aspirin use strictly to the peri-procedural phase.^9^ In this context, prasugrel and ticagrelor have demonstrated superior outcomes compared to clopidogrel in patients with ACS.^10^ Close clinical and laboratory monitoring should be performed in G6PD-deficient patients undergoing low-dose of aspirin.
